# Prophylactic ankle supports effects on time to stabilization, perceived stability and ground reaction force during lateral landing in female collegiate athletes with chronic ankle instability

**DOI:** 10.1186/s13102-021-00291-3

**Published:** 2021-06-03

**Authors:** Ali Yalfani, Zahra Raeisi

**Affiliations:** 1 Department of Sports Rehabilitation, Faculty of Sport Science, Bu-Ali Sina University, Hamedan, Iran; 2 Department of Sports Rehabilitation, Faculty of Sport Science, Arak University, Arak, Iran

**Keywords:** Kinesiotape, Lace-up brace, Lateral landing, Dynamic control

## Abstract

**Background:**

This study was designed to investigate effects of Kinesiotape (KT) with closed basket weave method and lace-up braces (LB) on the vertical time to stabilization, peak vertical ground reaction force (PvGRF), and time to PvGRF as well as perceived stability during lateral landing of participants with chronic ankle instability before and after fatigue.

**Methods:**

Thirty female college athletes with chronic ankle instability of three conditions (control, KT, and LB) performed lateral landing from a 30 cm high step on the plantar pressure platform pre and post fatigue.

**Results:**

The pre-test findings on the rearfoot, of LB indicated negatively increased the PvGRF force (F_2,58_=3.63, *P* = 0.04) and decreased the time to PvGRF (F_2,58_=4.67, *P* = 0.01). The Bonferroni post-hoc testing revealed LB condition increased the PvGRF than the control (*P* = 0.002) and KT (*P* = 0.038). Also, the post-hoc testing showed LB condition decreased the time to PvGRF force than the control (*P* = 0.05) and KT (*P* = 0.01). The LB negatively prolonged vertical time to stabilization in the forefoot (F_2,58_=6.74, *P* = 0.002) and rearfoot (F_2,58_=6.13, *P* = 0.004) after fatigue. The post-hoc testing revealed LB condition generated a slower vertical time to stabilization than the control and KT conditions (*P* ≤ 0.05). The use of KT had no positive effects as elevated the PvGRF in the forefoot post fatigue (F_2,58_=7.11, *P* = 0.002). The post-hoc test uncovered that KT augmented the PvGRF than control (*P* = 0.01) and LB (*P* < 0.001). On the other hand, using KT had psychological effects at pre-fatigue which resulting significantly greater in perceived stability compared to other conditions (F_2,58_=9.65, *P* < 0.001). The post-hoc test showed that using KT increased perceived stability than LB (*P* = 0.004) and control (*P* < 0.001). Moreover, perceived stability improved significantly in KT and LB compared to the control condition at the post-fatigue (*P* ≤ 0.001).

**Conclusions:**

Despite the positive psychological impact of the prophylactic ankle supports, there were no positive effect on the vertical time to stabilization, PvGRF, and time to PvGRF. Further studies are needed to distinguish the psychological and actual effects of prophylactic ankle supports on athletes with chronic ankle instability.

## Background

Lateral ankle sprains (LASs) are the most prevalent musculoskeletal injuries that occur among physically active individuals [1]. Such an injury is accompanied by a number of adverse effects, including significant athletic treatment costs; prolonged pain; absence from sport, work, and school; increased healthcare costs; decrease quality of life; increased odds ratio of recurrent ankle sprain; and premature arthritis [1–4]. Its high prevalence rate and resulting side effects have highlighted the need for specialists to accord urgent priority to the development of various prophylactic strategies intended to reduce the extent of ankle sprain injury. Current protective measures against the risk of ankle injury often encompass strength training for leg muscles, proprioceptive training, and the use of high-top shoes and prophylactic ankle supports (PASs) [5, 6].

PASs are classified mainly into ankle braces and tapes, each coming in different variants. They can effectively protect the ligament structure to prevent of ankle sprain by providing mechanical support and enhancing proprioception sense [7]. This prophylactic equipment is therefore frequently used by athletes with and without a previous history of LAS to prevent serious injury [1, 8].

Different injuries are caused by exercise activities and dominant movements in sports, among other factors. Volleyball and basketball, for example, account for 86 and 58 % of ankle injuries that occur during landing, respectively [9]. These physical traumas are potentially affected by the magnitude of the ground reaction force (GRF) acting on the body as it performs a landing task and the need to maintain the body’s perceived stability after initial foot contact with the ground [10]. A necessary requirement, then, is to minimize contact force during landing to reduce the force applied to ankle structures and thereby decrease damage to the ankles as the only body part that comes in contact with the ground during landing [8]. An athlete’s ability to effectively lessen landing effects is determined by the vertical GRF (vGRF) [11]^,^ whose peak value (i.e., PvGRF) has been investigated as a determinant of injury given that the large forces occurring at this moment are associated with injury or instability when combined with joint malalignment [12]. Another indicator explored in research is time to stabilization (TTS), which points to the ability of an individual to restore balance in a state wherein a shift in condition from dynamic to static motion occurs on the base of support [13]. The ability to stabilize rapidly after landing is considered a positive or prophylactic feature because injury mitigation is influenced by the effective maintenance of the body’s perceived stability after landing [12]. Finally, ankle injury can also be caused by fatigue. According to Tropp et al., more than 40 % of ankle injuries occur near the end of an activity and post-fatigue [14]. Fatigue reduces afferent inputs, decreases the ability to respond quickly to proprioceptive feedback, and ultimately increases postural oscillations and affects postural control [15, 16]. Fatigue is also one of the factors that directly affects vGRF and TTS after landing [17].

Although athletes frequently use PASs as protection against physical trauma, studies have presented contrasting results on PAS usage and their impact on GRF, time to peak GRF, and TTS. Some researchers, for instance, showed that the use of Kinesio tape differentially affects TTS and GRF [3, 6, 7, 9, 10, 18–20]. Hunt and Short found that taping increases self-esteem and reduces anxiety due to injury or re-injury [21]. In these studies, as well, the failure of PASs to influence participant performance and compare actual and psychological effects motivated researchers to put forward a placebo effect theory in regard to these instruments [22, 23].

Landing has been recognized as one of the most common causes of non-contact ankle sprain [24], with the direction of landing shown as an influencing factor for dynamic postural stability. In this respect, Liu et al. underscored the importance of perceived multidimensional stability tasks and suggested that lateral movements cause greater ankle injuries than do forward and vertical movements [13]. Despite the insights provided by such research, however, no study has been devoted to the effects of PASs on lateral landing before and after fatigue. This deficiency is worrisome because extant research involving forward jump landing tasks is insufficient to evaluate the causes and prevention of lower limb injuries. To fill this void, the current work conducted an experiment using lateral landing that imposes stress on the lateral and vulnerable parts of the ankle [13, 17]. Because the role of PASs in lateral landing and their effects on PvGRF, time to PvGRF, vertical TTS (vTTS), and perceived stability may differ from those of forward landing, this study investigated the effects of Kinesiotape (KT) and lace-up brace (LB) usage on the aforementioned variables before and after fatigue, with focus on female college student athletes with chronic ankle instability (CAI).

## Methods

### Participants

Thirty female collegiate athletes with CAI (mean age = 22.86 ± 2.01 years, mean mass = 56.76 ± 5.36 kg, mean height = 164.32 ± 4.4 cm, mean score of Cumberland Ankle Instability Tool (CAIT) = 20.2 ± 1.4) volunteered to participate in the study. The sample size was calculated by using GPower software, which was configured to run power analysis for a Cohen’s d effect size of 0.4, with alpha level of 0.05, and a test power of 0.95. A final sample of 30 was established to consider dropouts. The inclusion criteria, which were based on the standards approved by the International Ankle Consortium, were as follows: more than two incidences of ankle sprains or a greater need for medical treatment of the condition, feelings of fear and instability in ankle function, the collapse of the foot during physical activities, a score of ≤ 24 in the CAIT, and the confirmation of CAI via anterior drawer and talar tilt tests performed by an experienced physician [25]. The athletes recruited for participation should have had CAI on one foot only, with the affected leg as the dominant one. The participants were no history of ankle or knee injury in the past 3 months, as well as no history of surgery or fractures of the lower extremities; chronic diseases, such as patellofemoral pain syndrome; or apparent deformities, such as flat foot or high arch. The aim and procedures of the study were explained to the participants, who reviewed and voluntarily signed an informed written consent form. This project was approved by the local Ethics Committee (code IR.BASU.REC.1399.027) and also it was registered with an Iranian Registry of Clinical Trial (code IRCT20200204046368N3) and was performed according the Declaration of Helsinki.

### Instrumentation

A plantar pressure platform (FDM-S, Zebris Medical GmbH, Germany, 120 Hz acquisition frequency) composed of 2560 high-sensitivity sensors was used to record PvGRF, time to PvGRF, and vTTS during lateral landing.

### Experimental procedures

All participants were asked to report to the Sport Rehabilitation Research Laboratory for a single occasion. First, each participant performed warm-up exercises for 10 min.

**Fig. 1:**
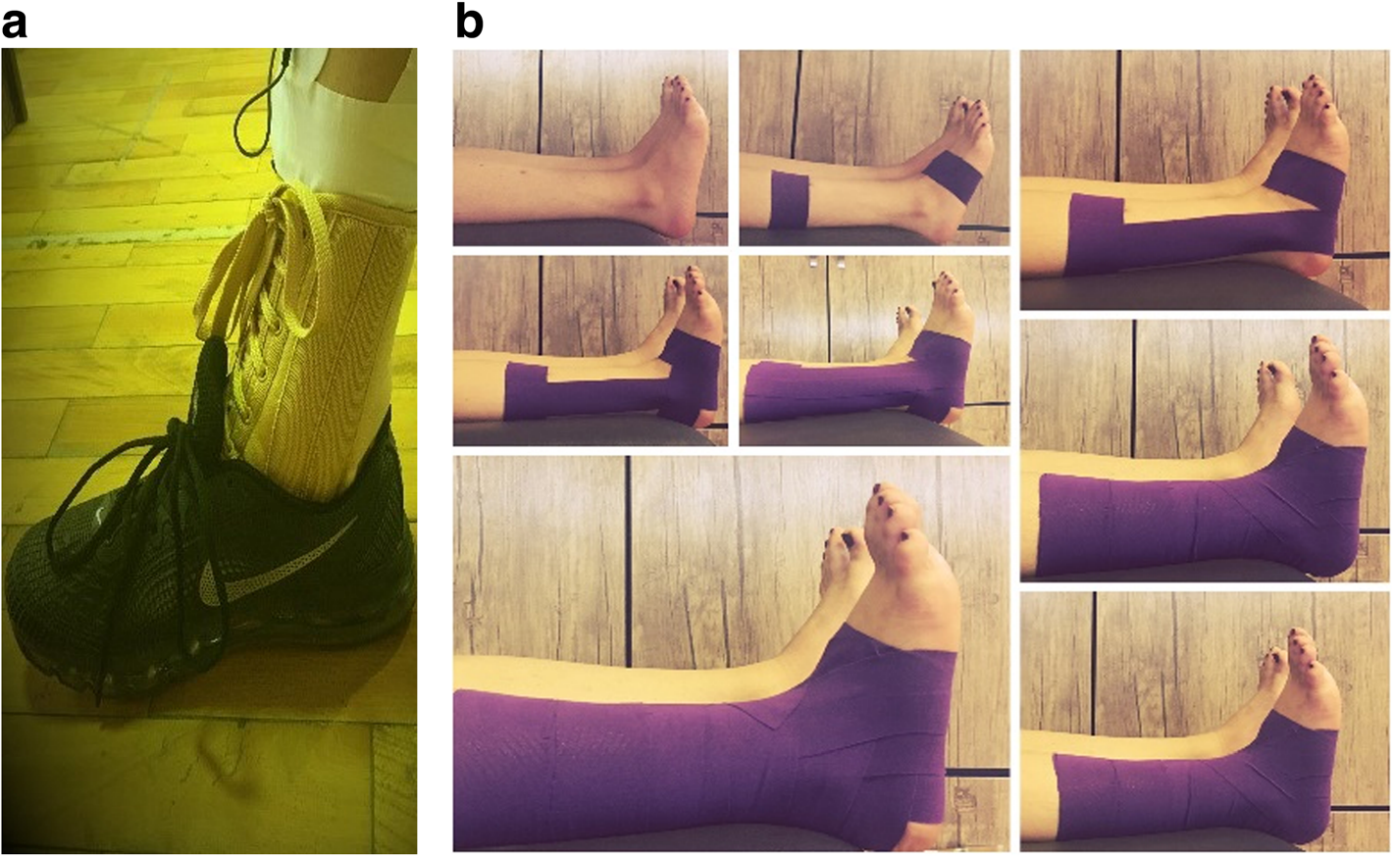
**a**. Sample of ankle lace-up brace. **b**. Sample of ankle closed basket weave pattern

The participant was tested under three conditions: The test limb was outfitted with a lace-up ankle brace (Model 4007, Oppo, USA) (Fig. 1a), wrapped in KT (Ares, Korea) in a complete closed basket weave pattern [26] (Fig. 1b), and left without a PAS (control). All the intervention applied by one certified athletic trainer to participants. The subject was then asked to perform a drop lateral landing task from a 30 cm high step that was positioned 15 cm from the center of a plantar pressure platform. To ensure that the task was correctly executed, the subject was instructed to place her non-dominant leg on the step while fully supporting her body weight. The participant was subsequently prompted to carry out drop lateral landing while looking forward without putting weight on the test limb as she landed on the platform and maintain her balance for 10 s after landing.

Each participant underwent three practice trials before the identification of the correct task was initiated. All of them performed three lateral landing tasks on the test limb under the three aforementioned conditions before and after fatigue and were wearing the same type of shoes (Air Max, Nike). Each participant attended in 3 testing session which in each session 1 of 3 conditions (KT, LB and control) was performed. A minimum of 48 and a maximum of 96 h of rest were given between sessions to all subjects [10]. The sequence of the tests (LB, KT, control) was determined by the participants randomly by lot.

### Fatigue protocol

The Bruce protocol was performed to induce fatigue by using h/p/cosmos mercury med treadmill. The Borg Rating of Perceived Exertion and Polar Pacemaker were used to determine time to fatigue and control heart rate, respectively. The fatigue protocol was terminated when the subjects reached a score of 17 on the Borg scale and registered 80 % of their maximum heart rate (age subtracted from 220). The subjects then performed the cool down phase for two minutes at their chosen speed. Immediately after fatigue, all the tests were repeated as part of the post-test.

### Psychological measure

A four-point Likert scale (1: *very unstable*, 2: *unstable*, 3: *stable*, 4: *very stable*) was used to assess the psychological effects of the examined PASs [22]. The subjects were requested to use this scale to indicate their perception of stability under each lateral landing condition before and after fatigue. They were prohibited from going over their prior responses to avoid influence from previously acquired scores. After completing all the tests, the subjects were asked to answer the following question: “If given a choice, which among the three conditions—the use of KT, outfitting with a LB, or the control condition—would you prefer to adopt during engagement in sports activities?”

### Data processing

**Fig. 2:**
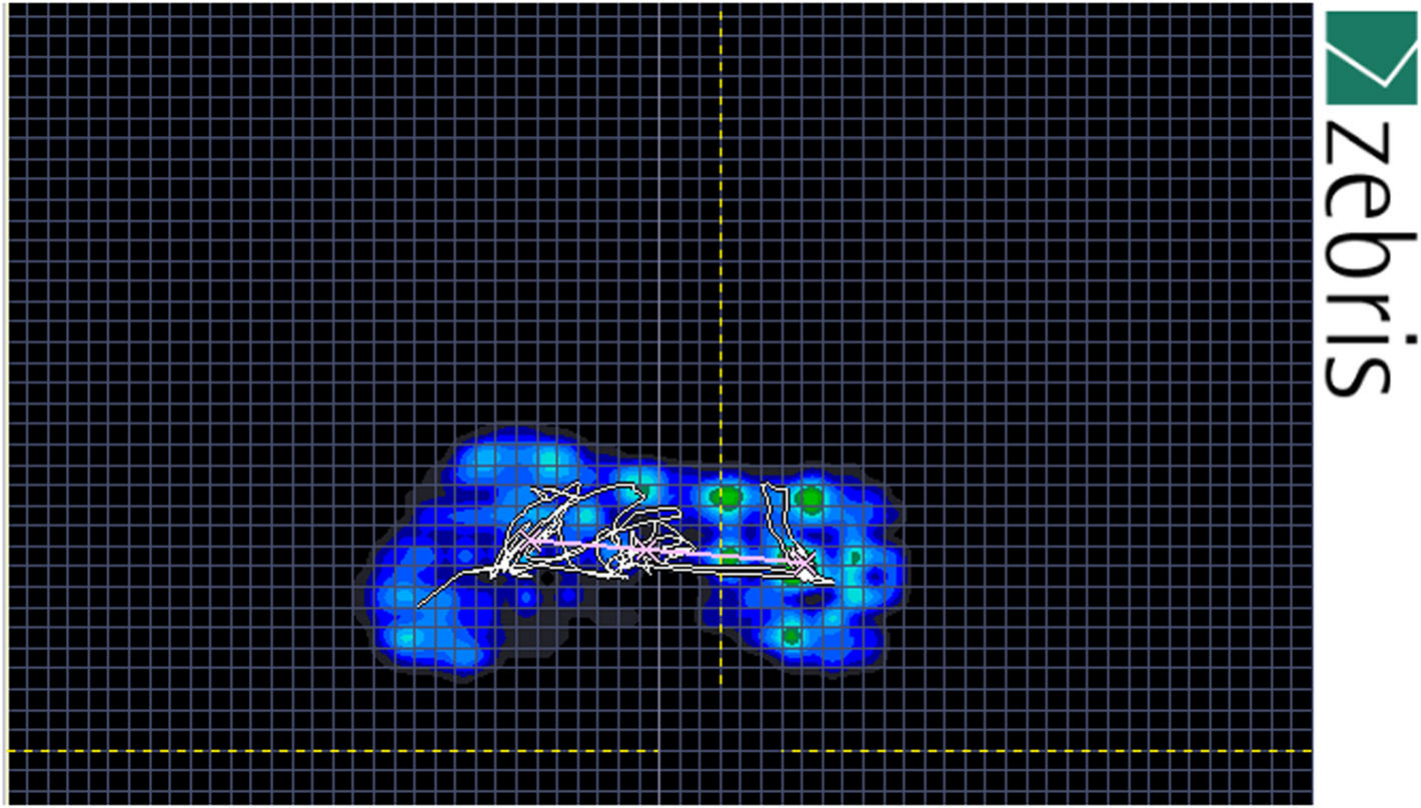
WinFDM-S software sample data

**Fig. 3:**
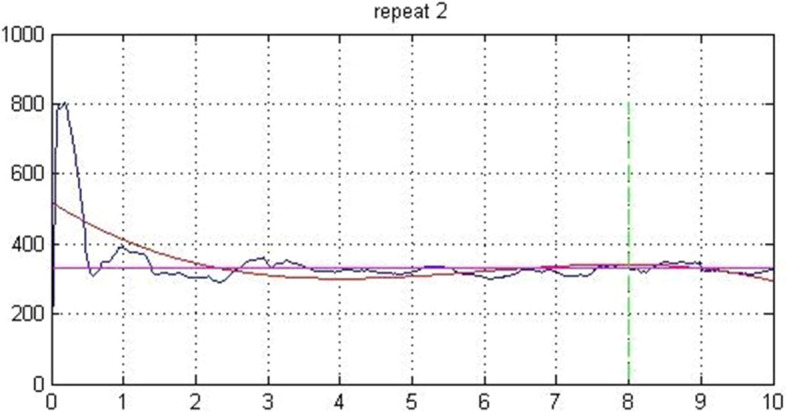
Example of calculation of vertical time to stabilization of 1 participant

Baseline vGRF data were obtained separately for the forefoot (two-thirds of foot length) and rearfoot (one-third of foot length) in each test using WinFDM-S software (Fig. 2). The trial data were then exported from the software, and the vTTS in each test was obtained using MATLAB (version R2010b, MathWorks, Natick, MA) following the procedure of Wright et al. [12]. Briefly, the data imported into MATLAB software were initially rectified and filtered with a 12 Hz second-order low-pass Butterworth filter. A normalized reference variable was calculated on the basis of the participants’ trial results; that is, the mean vGRF in the last two seconds of each trial (8–10 s) was ascertained. Subsequently, three standard deviations from the mean for a range of normal variations were calculated for each participant in all the trials. An unbounded third ordinal polynomial with a rectified force of 10 s after landing was fitted for each participant and each trial. The vTTS was defined as the point at which the unbounded third ordinal polynomial exceeded the range of variation occurring in the first trial (Fig. 3). The vTTS of the forefoot and rearfoot was calculated for each participant in the different test conditions.

PvGRF and time to PvGRF values were calculated in each test. Given that the test output was separately generated by software for the forefoot and rearfoot, the PvGRF and time to PvGRF derived on the basis of the forefoot and rearfoot data were labeled PvGRF1, time to PvGRF1, PvGRF2, and time to PvGRF2, respectively.

### Statistical analysis

The means and standard errors of the participants during the three lateral landing trials under each condition pre- and post-fatigue were subjected to statistical analysis. Four separate repeated-measures analyses of variance with two within-subject factors (condition and time) were conducted on PvGRF, time to PvGRF, vTTS, and perceived stability (dependent variables). A p value of 0.05 was considered statistically significant. The Bonferroni post-hoc test was carried out to determine statistically significant interactions, and the Statistical Package for the Social Sciences (version 21) was used to perform all the analyses.

## Results

Tables 1, 2 and 3 present the M ± SD of the studied variables.

**Table 1:** M±SD of variables examined in relation to the forefoot

Condition	Time	M±SD variables		
		vTTS(s)	PvGRF(N/cm^2^)	TT PvGRF (ms)
Fore foot				
Control	BF	1.61±0.92	984.02±295.48	0.17±0.16
	AF	1.44±0.93	939.89±208.76	0.12±0.03
Brace	BF	1.99±1.3	970.55±227.85	0.26±0.46
	AF	2.1±1.23	909.23±220.27	0.12±0.08
Tape	BF	1.85±1.17	936.04±220.34	0.1±0.03
	AF	1.02±0.57	975.79±211.8	0.1±0.02

Abbreviation: *BF* Before fatigue, *AF* After fatigue, *vTTS* Vertical time to stabilization, *vGRF* Vertical ground reaction force, *TT vGRF* Time to vertical ground reaction force

**Table 2:** M±SD of variables examined in relation to the rearfoot

Condition	Time	M±SD variables		
		vTTS(s)	PvGRF(N/cm^2^)	TT PvGRF (ms)
Rear foot				
Control	BF	1.36±1.05	515.45±92.92	1.04±1.57
	AF	1.24±1.12	582.61±133.66	0.85±1.15
Brace	BF	2.03±1.2	620.01±182.48	0.21±0.43
	AF	1.94±0.84	582.7±169.46	0.55±1.03
Tape	BF	1.74±1.09	574.87±159.91	1.29±2.23
	AF	0.98±0.94	600.98±162.16	0.83±1.08

Abbreviation: *BF* Before fatigue, *AF* After fatigue, *vTTS* Vertical time to stabilization, *vGRF* Vertical ground reaction force, *TT vGRFmax* Time to vertical ground reaction force

**Table 3:** M±SD of perceived stability score

Condition	Time	M±SD
Perception of stability		
Control	BF	3.2±0.32
	AF	2.75±0.6
Brace	BF	3.18±0.52
	AF	3.36±0.48
Tape	BF	3.72±0.47
	AF	3.80±0.44

Abbreviation: *BF* Before fatigue, *AF* After fatigue

### vTTS in the forefoot and rearfoot

A condition-by-time interaction (F_2,58_=6.74, *P* = 0.002, effect size = 0.2) occurred in relation to vTTS in forefoot. The post-hoc Bonferroni test revealed that outfitting with a LB resulted in a slower vTTS than that observed under the control (*P* = 0.01) and KT (*P* = 0.008) conditions after fatigue onset. The interaction between condition and time for vTTS in the rearfoot was significant (F_2,58_=6.13, *P* = 0.004, effect size = 0.19). The Bonferroni post-hoc test results indicated that being fitted with a LB generated a slower vTTS than that produced under the control (*P* = 0.009) and KT (*P* = 0.01) conditions post-fatigue.

### Perceived stability

The interaction between condition and time with respect to perceived stability (F_2,58_=9.65, *P* < 0.001, effect size = 0.27) was statistically significant. The post-hoc test showed that at pre-fatigue, using KT increased perceived stability to a level higher than that registered under the LB (*P* = 0.004) and control (*P* < 0.001) conditions. However, the latter two conditions generated no significant difference in perceived stability. The Bonferroni post-hoc test also showed that at post-fatigue, the use of KT increased perceived stability to a level greater than that observed under the other two conditions. After fatigue, fitting with a LB elevated perceived stability to a degree higher than that occurring under the controls (*P* < 0.001). In response to the general question about preference for landing conditions, 23 participants favored using Kinesio tape, and seven chose lace-up braces.

### PvGRF in the forefoot and rearfoot

Statistical analysis of time interaction of PvGRF in the forefoot indicated significantly differences between the three conditions (F_2,58_=7.11, *P* = 0.002, effect size = 0.21). The post-hoc test uncovered that at post-fatigue, using KT augmented the PvGRF at a degree exceeding that under the control (*P* = 0.01) and LB (*P* < 0.001) conditions. The condition-by-time interaction as regards PvGRF in the rearfoot also significantly differed across the interventions (F_2,58_=3.63, *P* = 0.04, effect size = 0.12). The pre-fatigue LB condition increased the PvGRF to an extent greater than that occurring under the control (*P* = 0.002) and KT (*P* = 0.038) conditions.

### Time to PvGRF in the forefoot and rearfoot

The results on the forefoot showed that no condition-by-time interaction as regards time to PvGRF occurred (F_2,58_=2.63, *P* = 0.1, effect size = 0.08) but that such an interaction was statistically significant with respect to the rearfoot (F_2,58_=4.67, *P* = 0.01, effect size = 0.15). The Bonferroni post-hoc test results revealed that at pre-fatigue, the use of LB decreased the time to PvGRF to a level lower than that registered under the controls (*P* = 0.05) and the use of KT (*P* = 0.01).

## Discussion

The present study was designed to investigate effects of KT with closed basket weave method and LB on the vTTS, PvGRF, and time to PvGRF in the forefoot and rearfoot as well as perceived stability during lateral landing performed by female college athletes with CAI before and after fatigue.

### vTTS and psychological effects on perceived stability

TTS is commonly examined in research on postural stability and the functioning of lower extremity joints, such as ankles and knees. It is defined as the time required for an individual to return to baseline values from an unstable position [27]. The results of this study showed that at pre-fatigue, the PASs had no effect on vTTS in the forefoot and rearfoot during lateral landing, whereas at post-fatigue, the use of LB negatively affected vTTS in the forefoot and rearfoot and generated a vTTS longer than that observed under the control and KT conditions. PASs are widely used among athletes to prevent injury, and the use of tape and braces has attracted the attention of many researchers [2, 3, 6, 10]. Various studies on KT derived different effects, including increased skin blood flow during exercise, modified lymphatic circulation, support for ligaments and tendons, and a stimulated subcutaneous skin receptor mechanism; these effects enhance the activity of mechanoreceptors through a feedback mechanism and improve joint performance [5, 7]. Two broad theories have been used to explain the mechanism of brace efficacy: the first features passive mechanical support, and the second revolves around the improvement of sensorimotor function through increased stimulation of cutaneous receptors and joint mechanoreceptors [28].

Our hypothesis was that a brace model with support for lateral parts of the ankle can help control this body part during lateral landing and thus improve balance. Ankle stability was predicted to increase because of the eight-like shape and heel-lock structure used in the completed closed basket weave taping method. In spite of the advantages attributed to these PASs in the literature and in contrast to the hypothesis that we formulated, we did not observe a significant difference in vTTS during lateral landing with KT usage; meanwhile, at post-fatigue, the use of LB increased vTTS in the forefoot and rearfoot. Studies have shown that fatigue clearly affects the biomechanics of landing on one foot and that the risk of injury is greater after fatigue. Fatigue may alter neuromuscular control and diminish the body’s ability to maintain stability [6, 11], thus driving the use of TTS in evaluating the impact of fatigue on proprioception and neuromuscular control. An increase in this variable indicates the body’s delayed response to stability and difficulty in postural control during landing [6, 27].

According to the results, LB usage did not improve vTTS and increased vTTS post-fatigue, which can increase the risk of injury. Brace weight potentially weakens postural control during fatigue and increases vTTS during lateral landing. Significant results were derived as regards psychological effects on the perceived stability of the participants. Unlike the LB and control conditions, KT usage resulted in greater perceived stability pre-fatigue. Such stability did not differ significantly under the LB and control conditions. Despite the increase in perceived stability during landing with KT use, the vTTS did not decrease significantly in this condition. Consistent with these results, Hunt and Short and Gear et al. reported an improvement in subjects’ feelings of stability and self-confidence in performing functional tests with KT fitting, even though no significant difference was found in the participants’ performance [21, 22]. In our study, the participants felt that the ankle was more stable post-fatigue when they used a PAS in executing landing than when no support was employed. In addition to the tape, the LB also significantly differed from the controls in terms of improvement to perceived stability.

The vTTS results increased with LB usage during lateral landing. Sawkins et al. stated that if athletes believe that PASs protect them from injury, they may participate in an activity more confidently [23]. In the present work, the increase in vTTS with LB usage at post-fatigue may have enhanced the participants’ sense of stability—an outcome that did not arise before the onset of fatigue. According to expectancy theory, athletes rely on the effects of PASs in preventing injury. As a result, inducing the belief that a placebo is effective and increasing perceived stability are easy, as asserted by Sawkins et al. [23]. However, the use of PASs seems to result in less precise lateral landing owing to the creation of a false feeling of safety and an increase in false self-confidence. This can increase the risk of injury, especially during fatigue, in more difficult situations, such as races and competitions or the performance of complex and high-speed tasks.

A notable finding in this work was that all the 30 subjects with CAI preferred using the PASs as supports in performing landing, with 23 favoring KT for the increased sense of ankle stability that it provided, and seven preferring to use LB.

### PvGRF and time to PvGRF

At pre-fatigue, using LB increased PvGRF in the rearfoot but decreased the time to PvGRF. The use of KT augmented PvGRF in the forefoot during lateral landing after fatigue. PvGRF is a pivotal and desirable variable for evaluating landing because it eases measurement and generates accurate results [8]. It can also indicate an athlete’s ability to effectively reduce landing effects. The lower PvGRF show the better landing strategy; strong force can lead to injuries to the ankle and knee joints [11]. PASs are primarily intended to limit the excessive inversion of the ankle and foot complex while allowing normal plantar flexion and dorsiflexion to maintain function. However, studies showed that the range of motion (ROM) of plantar flexion and dorsiflexion will also be limited when an ankle brace or tape is used [3, 7, 10, 29]. Decreasing the normal ROM of the ankle can affect the entire lower extremity and normal movement patterns and thereby weaken the body’s ability to absorb energy upon landing and impose greater GRF on the body [10]. Damage to structures such as the subchondral bone, cartilage, and soft tissue may also occur as a result of increased GRF [3].

Studies demonstrated that the use of PASs not only influences the ROM of joints but also reduces muscle activity, which may diminish the auxiliary role of some muscles in minimizing body acceleration during landing [28]. Fatigue is an integral part of physical activity. When it occurs, reaction times against external stimuli are delayed, and the likelihood of injury increases [27]. This risk of injury is exacerbated on initial contact because the body at this stage cannot move within an ROM to allow contact forces to be absorbed through active structures (such as muscles) [27, 29]. Previous studies reported an increase in PvGRF in forward drop landing with decreasing joint ROM and leg muscle activity due to PAS use [3, 20, 29]. Our results also indicated that PvGRF increased before and after fatigue in rear foot under the use of LB and KT, respectively. Similarly, the use of KT increased PvGRF in the forefoot post-fatigue. These results are consistent with those of Cordova et al. [29] and Distefano et al. [3] but inconsistent with those of Hodgson et al. [20] Note that the task performed in the present study was lateral landing, whereas that in most previous works was forward landing.

The findings likewise illustrated that ankle bracing reduced the time to PvGRF in the rearfoot to levels lower than those occurring under the control and KT conditions. The increasing PvGRF and decreasing time to PvGRF during lateral landing under LB usage suggested that under this condition, musculoskeletal structures are affected by greater loads imposed at a shorter time [10]. These changes also implied that the ankle’s ability to absorb energy decreases when certain PASs are used; this effect, in turn, increases the load imposed on proximal joints, including the knee [10]. In this regard, Cordova et al. showed that some ankle stabilizers impair the optimal performance of this joint in absorbing contact by restricting the ROM of the ankle [29]. Our results are consistent with those derived by Henderson et al.[9] and Hodgson et al. [20] on the increase in PvGRF during landing with braces. They are also compatible with the findings of Riemann et al. [10] on the reduction of time to PvGRF.

Because the LB used in this study covers a large area of the soles of the foot and provides support to the lateral parts of the ankle, its use likely reduced the ROM of the rearfoot and may have decreased the time to PvGRF in the rearfoot by shortening its contact with the ground. Contrary to studies on forward landing drops that found a PvGRF2 larger than PvGRF1, the current research uncovered a PvGRF2 larger than PvGRF1 during lateral landing and a PvGRF1 greater than PvGRF2 at all landing conditions before and after fatigue.

The literature discussed the effects of general fatigue on balance, which is why a general fatigue protocol was used in the present study. Nevertheless, a different mechanism and effect may arise with regard to functional fatigue. Given that only a lateral landing test was performed on the injuried leg, no data on the other leg was derived for comparison in regard to PAS effects. Findings may also differ depending on task type. Finally, the same shoes were used by all the participants for matching in the test, but using other types of footwear may generate different results.

## Conclusions

Our results showed that the examined PASs had no positive effect on vTTS, PvGRF, and time to PvGRF in the forefoot and rearfoot despite their positive psychological influence on the participants’ perceived stability during lateral landing. Because the results obtained for the forefoot and rearfoot were separately accurate, PvGRF in the rearfoot was not necessarily larger than PvGRF in the forefoot during task performance. These values appear to be directly related to individual landing techniques and type of task performed.

Given that only lateral landing was examined in this work, the use of KT and LB in other tasks may produce varying outcomes. Further study should be directed toward distinguishing the actual and psychological effects of PASs specially after fatigue in other tasks.

## Data Availability

The datasets used and/or analyzed during the current study are available from the corresponding author on reasonable request.

## Abbreviations

LASs: Lateral ankle sprains
PASs: Prophylactic ankle supports
GRF: Ground reaction force
vGRF: Vertical ground reaction force
TTS: Time to stabilization
KT: Kinesiotape
LB: Lace-up brace
CAI: Chronic ankle instability
CAIT: Cumberland Ankle Instability Tool
PvGRF: peak vertical GRF
BF: Before fatigue
AF: After fatigue
vTTS: Vertical time to stabilization
TT vGRF: Time to vertical ground reaction force
ROM: Range of motion
